# Dr. Joseph W. Keene

**Published:** 1902-03

**Authors:** 


Dr. Joseph W. Keene, of Fallbrook, Cal., died at his home January 30, 1902, from apoplexy, aged 54 years. He was a graduate of Harvard University, class of 1878, and resided in Buffalo for several years. He was a man of accomplishment, and enjoyed the esteem of his colleagues.

